# Effect of glycemic control and glucose fluctuation on in-hospital adverse outcomes after on-pump coronary artery bypass grafting in patients with diabetes: a retrospective study

**DOI:** 10.1186/s13098-023-00984-4

**Published:** 2023-02-14

**Authors:** Hongzhao You, Xiaopei Hou, Heng Zhang, Xiaojue Li, Xinxing Feng, Xin Qian, Na Shi, Rong Guo, Xuan Wang, Hansong Sun, Wei Feng, Guangwei Li, Zhe Zheng, Yanyan Chen

**Affiliations:** 1 Endocrinology Center, Fuwai Hospital, National Center for Cardiovascular Diseases, Chinese Academy of Medical Sciences and Peking Union Medical College, No.167 North Lishi Road, Xicheng District, Beijing, 100037 China; 2 Department of Cardiovascular Surgery, Fuwai Hospital, National Center for Cardiovascular Diseases, National Clinical Research Center of Cardiovascular Diseases, State Key Laboratory of Cardiovascular Disease, Chinese Academy of Medical Sciences and Peking Union Medical College, Beijing, China

**Keywords:** Diabetes, Coronary artery bypass grafting, Cardiopulmonary bypass, Blood glucose fluctuation, Adverse outcome

## Abstract

**Background:**

The optimal glycemic control level in diabetic patients undergoing coronary artery bypass grafting (CABG) with cardiopulmonary bypass (On-Pump) remains unclear. Therefore, this study aimed to investigate the effect of different blood glucose control levels and glucose fluctuations on in-hospital adverse outcomes in diabetic patients undergoing on-pump CABG.

**Method:**

A total of 3918 patients with diabetes undergoing CABG were reviewed in this study. A total of 1638 patients were eligible for inclusion and were categorized into strict, moderate and liberal glucose control groups based on post-operative mean blood glucose control levels of  < 7.8 mmol/L, from 7.8 to 9.9 mmol/L and ≥ 10.0 mmoL/L, respectively. The primary endpoint was defined as a composite endpoint including in-hospital all-cause mortality and major cardiovascular complications. The secondary endpoint was defined as major cardiovascular complications including acute myocardial infarction, strokes and acute kidney injuries. To determine the associations between blood glucose fluctuations and adverse outcomes, patients with different glycemic control levels were further divided into subgroups according to whether the largest amplitude of glycemic excursion (LAGE) was ≥ 4.4 mmol/L or not.

**Results:**

A total of 126 (7.7%) patients had a composite endpoint. Compared with moderate control, strict glucose control was associated with an increased risk of the primary endpoint (adjusted OR = 2.22, 95% CI 1.18–4.15, p = 0.01) and the secondary endpoint (adjusted OR = 1.95, 95% CI 1.01–3.77, p = 0.049). Furthermore, LAGE ≥ 4.4 mmol/L was significantly associated with the primary endpoint (adjusted OR = 1.67, 95% CI 1.12–2.50, p = 0.01) and the secondary endpoint (adjusted OR = 1.75, 95% CI 1.17–2.62, p = 0.01),respectively. Patients with LAGE ≥ 4.4 mmol/L had significantly higher rates of the composite endpoint and major vascular complications in both the strict-control (the primary endpoint, 66.7% vs 12.4%, p = 0.034, the secondary endpoint, 66.7% vs 10.3%, p = 0.03) and moderate-control groups (the primary endpoint, 10.2% vs 6.0%, p = 0.03, the secondary endpoint, 10.2% vs 5.8%, p = 0.02).

**Conclusions:**

After On-Pump CABG patients with diabetes, strict glucose control (< 7.8 mmol/L) and relatively large glucose fluctuations (LAGE ≥ 4.4 mmol/L) were independently associated with in-hospital adverse outcomes.

**Supplementary Information:**

The online version contains supplementary material available at 10.1186/s13098-023-00984-4.

## Background

Coronary artery bypass grafting (CABG) is an effective method of revascularization in diabetic patients with coronary artery disease (CAD). However, in patients with pre-existing diabetes mellitus, cardiac surgery outcomes are poor and unpredictable [1]. Moreover, uncontrolled blood glucose levels in diabetic patients are associated with adverse perioperative outcomes. To maintain favorable clinical outcomes, attention to perioperative glycemic control is crucial. Guidelines recommend that blood glucose be controlled under 10.0 mmol/L to reduce perioperative cardiovascular events [2]. A relatively tight glucose level (4.4–6.1 mmol/L) was initially recommended to reduce short-term adverse events in hospitalized diabetic patients after CABG [3]. However, an increasing number of studies have shown that tight glucose control may not be as beneficial as originally thought [4, 5], particularly in patients undergoing On-Pump CABG. Blood glucose fluctuations were more common in patients with strict blood glucose control, resulting in worse outcomes [6]. Our previous study showed that strict glucose control (< 7.8 mmol/L) was associated with an increased risk of in-hospital mortality in patients with diabetes [7]. Postoperative glycemic control in diabetic patients undergoing On-pump CABG is challenging for physicians because it depends on both the physicians’ clinical experience and the patients’ response to hypoglycemic therapy [8]. However, precise and optimal glycemic control is clinically needed by this patient population.

This study explores, for the first time, the association between different glycemic control levels and in-hospital outcomes in diabetic patients after On-Pump CABG, including the impact of blood glucose fluctuations on adverse patient outcomes.

## Methods

### Study population

**Fig. 1:**
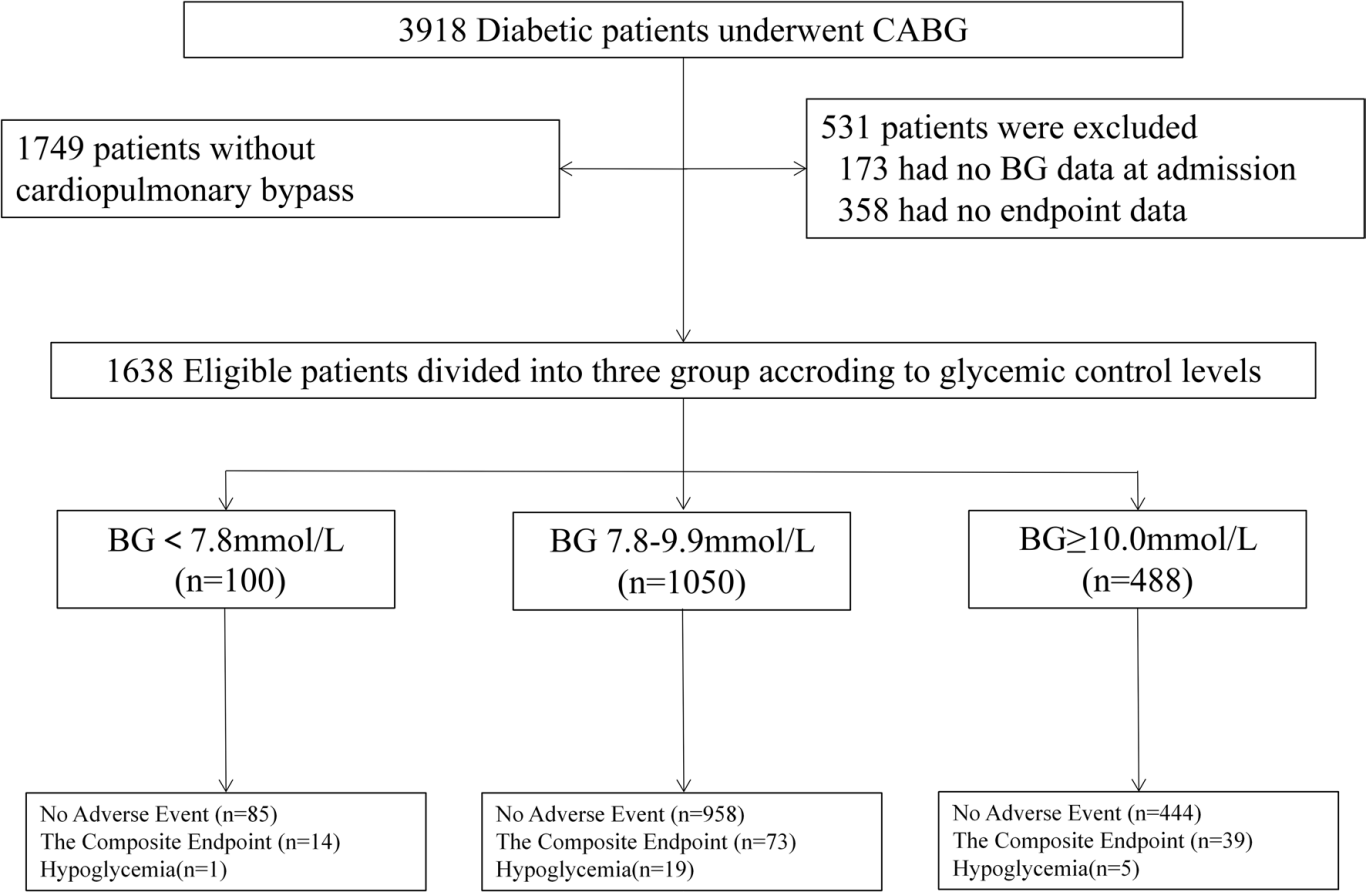
Study Flow Chart

We first extracted and identified the medical records of 3198 diabetic patients undergoing CABG between January 2011 and December 2014 in Fuwai Hospital. Data including patient demographic and clinical characteristics, as well as in-hospital outcomes, were collected by trained physicians via chart review. Figure 1 shows a flowchart of the study. Diabetes was defined as preoperative glycosylated hemoglobin (HbA1c) ≥ 47.5 mmol/mol(6.5%), fasting blood glucose ≥ 7.0 mmol/L or a documented history of diabetes [9]. Cardiac surgical procedures and extracorporeal circulation support schemes were decided by a multidisciplinary team, depending on the patient’s lesions, presurgical evaluations, complications, and doctors’ experience. A total of 531 patients were excluded because of missing blood glucose values or adverse events during the admission period. Overall, 1638 eligible patients undergoing On-Pump CAB were included in this study.

During the perioperative period, diabetic patients with a random blood glucose ≥ 180 mg/dL (10.0 mmol/L) received a continuous intravenous insulin infusion or multiple subcutaneous insulin injections with a target blood glucose level of 7.8–10 mmoL/L [10, 11]. This follows current diabetes management guidelines. Blood glucose measurements were obtained through arterial catheters or capillary blood glucose every two hours in patients receiving intravenous insulin treatment and every four hours in patients who did not receive insulin therapy. The mean blood glucose (MBG) was calculated and represented as the daily average blood glucose after CABG during hospitalization. This method of BG data collection has been previously described [12]. The largest amplitude of glycemic excursion (LAGE), a reliable indicator of blood glucose fluctuations, corresponded to the difference between the maximum and minimum blood glucose levels in a day. LAGE ≥ 4.4 mol/L was identified as an independent risk factor of peripheral vascular disease in patients with type 2 diabetes [13]. Therefore, we used this cut-off value in our study.

To explore the association between clinical outcomes and glucose control, patientsʼ glycemic control status was categorized into the following three groups according to MBG after CABG, as we have previously reported [7]: strict-control group, MBG < 7.8 mmol/L; moderate-control group, MBG from 7.8 to 9.9 mmol/L; and liberal-control group, MBG ≥ 10.0 mmol/L.

Other pharmacological treatments were recommended based on current clinical practice, including angiotensin-converting enzyme inhibitors, beta-blockers and statins.

### Clinical outcomes

The primary endpoint was a composite outcome of major cardiovascular complications and all-cause in-hospital mortality. Major vascular complications, as the secondary endpoint, included postoperative acute myocardial infarction, stroke or acute renal failure. All of the outcome components were determined according to the definitions provided by the American Heart Association and Society of Thoracic Surgeons [14, 15]. Acute myocardial infarction was defined as a cardiac biomarker level (creatine kinase-MB, cardiac troponin T, cardiac troponin I or lactate dehydrogenase) more than ten times the upper reference limit within 48 h after CABG, with the presence of at least one of the following: new left bundle branch blocks; new pathological Q waves; evidence of new loss of myocardia or regional wall motion abnormalities or angiographic evidence of new graft or native coronary occlusion. Strokes were defined as a new onset of neurologic deficits originating from vascular brain lesions and lasting more than 24 h. Acute renal failure was defined as serum creatinine higher than 353.6 µmol/L or three times higher than the preoperative level.

### Statistical analysis

For baseline characteristics, continuous variables were expressed as mean ± SD and compared using ANOVA or Kruskal–Wallis tests and categorical variables were expressed as frequencies (percentages) and compared using chi-squared tests or Fisher’s exact tests. Univariate logistic regression models were used to determine the relationship between the glycemic control level and the pre-defined primary and secondary endpoints for diabetic patients after On-pump CABG (ORs with 95% confidence intervals). Two multivariate logistic regression models were further constructed, of which Model 1 adjusted the odds ratios for age and gender, while Model 2 adjusted the odds ratios for age, sex, smoking, systolic blood pressure, low-density lipoprotein cholesterol, glycosylated hemoglobin, chronic renal failure, congestive heart failure, previous myocardial infarction and previous peripheral vascular diseases. The variables were previously reported as established risk factors affecting the prognosis of diabetic patients and were simultaneously selected into multivariate logistic models. The adjusted odds ratios, together with corresponding 95% confidence intervals, were reported. All statistical analyses were performed using a two-sided significance level of 0.05. Univariate and multivariable logistic regression models were also used to determine the relationship between LAGE and the pre-defined primary and secondary endpoints for diabetic patients after On-Pump CABG (ORs with 95% confidence intervals). The statistical analysis software used was SAS^®^ 9.4 (SAS Institute, Cary, NC, USA).

## Results

### Baseline characteristics

A total of 1638 diabetic patients who underwent CABG were included in our study. Overall, the mean age of all patients was 60.9 ± 8.6 years and 25.5% of them were females. Compared with the moderate-control group, the liberal-control group contained more female patients and had a higher BMI, and the strict-control group contained more smokers. Meanwhile, there was no difference in laboratory results or complications (all p ≥ 0.05) among the three groups (Table 1).

**Table 1:** Baseline Characteristics of patients stratified by glycemic control levels

Variable	All patients (n = 1638)	Liberal control (n = 488)	Moderate control (n = 1050)	Strict control (n = 100)
Demographic data				
Age,Yrs	60.9 ± 8.6	61.1 ± 8.5	60.8 ± 8.5	61.4 ± 8.9
Gender (female,%)	417 (25.5)	146 (28.9)*	253 (24.1)	18(18.0)
Body mass index, kg/m2	26.1 ± 3.2	26.4 ± 3.1*	26.0 ± 3.2	25.6 ± 3.6
Smoker (n, %)	654 (39.9)	194 (40.0)	409 (39.0)	51 (51.0)*
Laboratory data				
HbAlc,%	7.6 ± 1.3	7.9 ± 1.3*	7.2 ± 2.2	7.1 ± 2.2
TG,mmol/L	1.8 ± 1.1	1.9 ± 1.2*	1.8 ± 1.0	1.6 ± 0.7
TC,mmol/L	4.1 ± 1.1	4.2 ± 1.1	4.1 ± 1.1	4.1 ± 1.0
HDL, mmol/L	1.0 ± 0.3	1.0 ± 0.3	1.0 ± 0.3	1.0 ± 0.3
LDL, mmol/L	2.5 ± 0.9	2.5 ± 0.9	2.5 ± 1.0	2.5 ± 0.8
Complication				
Hypertension (%)	1083 (66.1)	327 (67.0)	690 (65.7)	66 (66.0)
Dyslipidemia (%)	1017 (62.1)	288 (59.0)	664 (63.2)	65 (65.0)
Chronic kidney disease (%)	3 (0.2)	0 (0)	3 (0.3)	0 (0)
Previous MI (%)	485 (29.6)	159 (32.6)	293 (27.9)	33 (33.0)
Previous stroke (%)	195 (11.9)	58 (11.9)	125 (11.9)	12 (12.0)
Previous PAD (%)	247 (15.1)	78 (16.0)	159 (15.1)	10 (10.0)
Previous HF (%)	310 (18.9)	98 (20.1)	199 (19.0)	13 (13.0)

Values are mean ± SD

HbAlc, Glycosylated Hemoglobin; TG, triglyceride; TC total cholesterol; HDL, high-density lipoprotein; LDL, low-density lipoprotein; MI, myocardial infraction; PAD, peripheral arterial disease; *P < 0.05 is significant,comparing with the moderate control group

### Impact of different glycemic control levels on the in-hospital mortality and major vascular complications

**Fig. 2:**
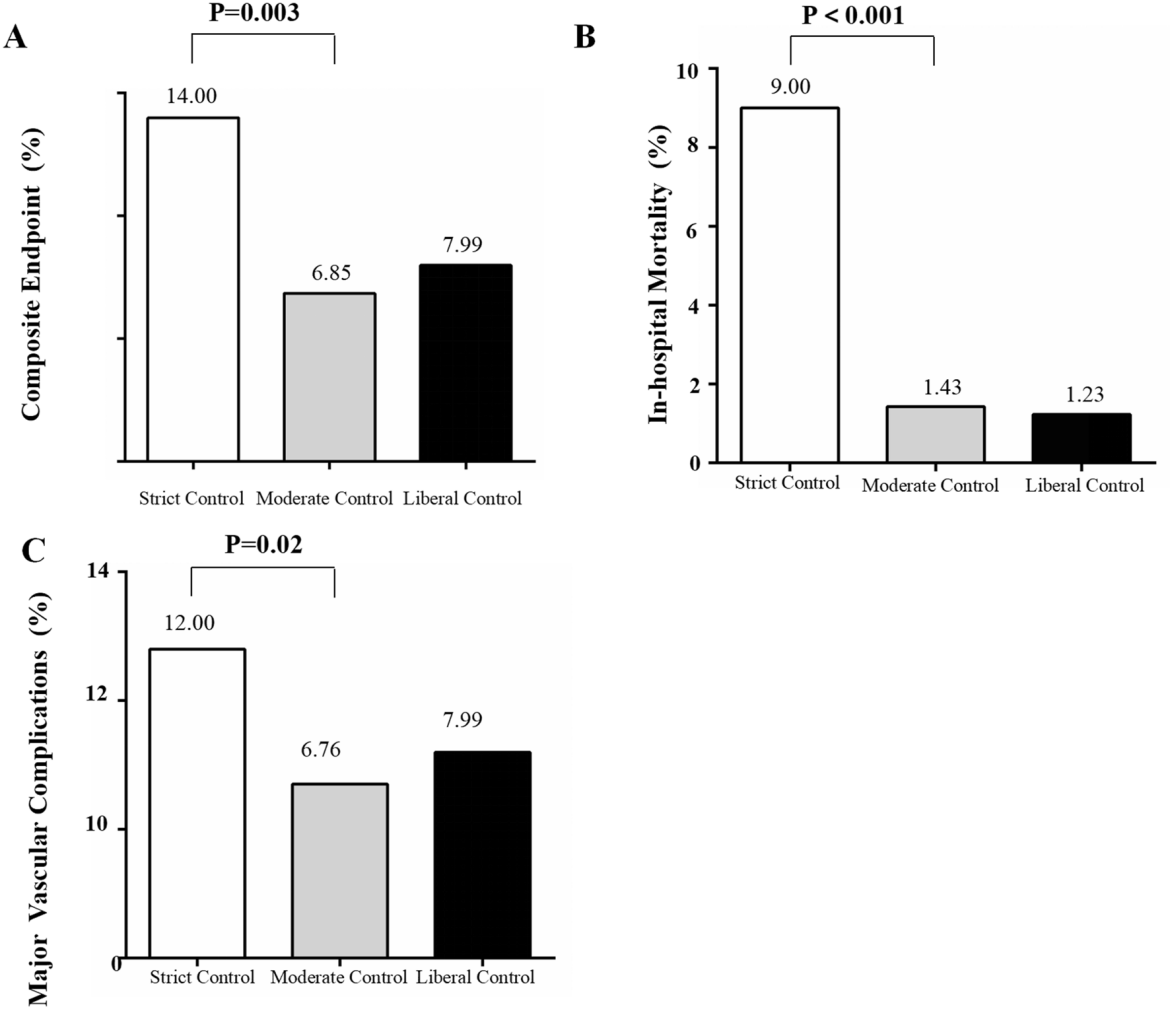
Outcomes rates after On-pump CABG by glycemic control levels for patients with diabetes. **a** composite endpoint. **b **in-hospital mortality. **c**major vascular complications

During admission, 126 patients met the composite endpoint. Compared with the moderate-control group, the strict-control group showed higher rates of the composite endpoint (14.00% vs 6.85%, p = 0.003), in-hospital mortality (9.00% vs 1.43%, p < 0.001) and major vascular complications (12.00% vs 6.76%, p = 0.02) (Fig. 2 and Additional file 1: Table S1).

We further explored the association between glycemic control levels and in-hospital adverse outcomes. As shown in Table 2, after adjusting for age, sex, BMI, smoking, low-density lipoprotein cholesterol, chronic renal failure, congestive heart failure, previous myocardial infarction and peripheral vascular diseases, strict glycemic control, compared with moderate glycemic control, was associated with an increased risk of the composite endpoint (adjusted OR = 2.22, 95% CI 1.18–4.15, p = 0.01), in-hospital mortality (adjusted OR = 7.57, 95% CI 2.89–19.87, p < 0.01) and major vascular complications (adjusted OR = 1.95, 95% CI 1.01–3.77, p = 0.049).

**Table 2:** Association of glycemic control levels with the composite endpoint in diabetic patients undergoing CABG with CPB

Glycemic control	Crude Odd Ratio	95% Confidence Interval	p-value	Model 1	95% confidence Interval	P-value	Model 2	95% confidence Interval	p-Value
The composite end point									
Moderate control	References								
Strict control	2.18	1.18–4.02	0.01	2.13	1.15–3.95	0.02	2.22	1.18–4.15	0.01
Liberal control	1.16	0.78–1.74	0.74	1.15	0.77–1.73	0.49	1.14	0.76–1.72	0.53
In-hospital mortality									
Moderate control	References								
Strict control	6.81	2.90–15.98	< 0.01	6.31	2.65–15.01	< 0.001	7.57	2.89–19.87	< 0.01
Liberal control	0.86	0.33–2.23	0.75	0.82	0.31–2.15	0.68	0.84	0.31–2.27	0.73
Major vascular complications									
Moderate control	References								
Strict control	1.88	0.98–3.60	0.06	1.89	0.99–3.59	0.05	1.95	1.01–3.77	0.049
Liberal control	1.20	0.80–1.80	0.38	1.20	0.80–1.81	0.37	1.77	0.77–1.77	0.46

Adjusted odds ratios for the relationships between glucose control and in-hospital mortality and major complications. The multivariable logistic regression regression model 1 for odds ratios included adjustments for age and gender. The multivariable logistic regression regression model 2 for odds ratios included adjustments for age, sex, smoking, systolic blood pressure, lowdensity lipoprotein cholesterol, glycosylated hemoglobin, chronic renal failure, congestive heart failure, previous myocardial infarction, previous peripheral vascular disease

### Association between post-operative blood glucose fluctuation and composite endpoint

**Fig. 3:**
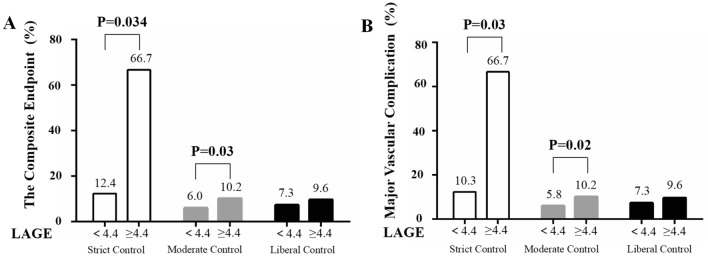
Comparison of outcomes rates stratified by the levels of largest amplitude of glycemic excursions at different blood glucose control levels in patients with diabetes undergoing On-pump CABG. **a**, composite endpoint. **b**,major vascular complications

To examine whether LAGE is an independent risk factor for in-hospital adverse events for diabetic patients undergoing On-Pump CABG, adjustments were made for age, sex, BMI, smoking, low-density lipoprotein cholesterol, chronic renal failure, congestive heart failure, previous myocardial infarction, and peripheral vascular diseases. Following the above adjustments, with LAGE ≥ 4.4 mol/L, significant associations remained between the composite endpoint (adjusted OR = 1.67, 95% CI 1.12–2.50, p = 0.01) and major vascular complications (adjusted OR = 1.75, 95% CI 1.17–2.62, p = 0.01). We further investigated the association between blood glucose fluctuations and in-hospital adverse outcomes at different blood glucose control levels. Hospital outcomes comparisons are shown in Fig. 3. Patients with LAGE ≥ 4.4 mmol/L had significantly higher rates of the composite endpoint and major vascular complications in both the strict-control (the composite endpoint, 66.7% vs 12.4%, p = 0.034, major vascular complications, 66.7% vs 10.2%, p = 0.03) and moderate-control groups (the composite endpoint, 10.2% vs 6.0%, p = 0.03, major vascular complications, 10.2% vs 5.8%, p = 0.02).

## Discussion

In the present study, we investigated the associations between different glycemic control levels and in-hospital adverse outcomes in diabetic patients who underwent On-Pump CABG. In these patients, strict glycemic control (< 7.8 mmol/L) was associated with increased risks of the composite endpoint and major vascular complications, compared with moderate glycemic control (7.8–9.9 mmol/L). Meanwhile, we found that abnormal blood glucose fluctuations also impacted in-hospital adverse outcomes. In the strict-control and moderate-control groups, patients with LAGE ≥ 4.4 mmol/L had higher rates of both the composite endpoint and major vascular complications, compared with patients with LAGE < 4.4 mmol/L. Additionally, LAGE ≥ 4.4 mmol/L was an independent risk factor for both the composite endpoint and major vascular complications.

### Blood glucose control level of diabetic patients undergoing on-pump CABG

Hyperglycemia in the perioperative period is widely recognized as a robust risk factor for adverse events in patients after CABG, especially in the early stage of hospitalization [16]. Appropriate glycemic control can reduce perioperative adverse events and improve prognosis [17]. For diabetic patients, an excessively liberal glycemic control (> 10 mmol/L) can increase the risk of in-hospital adverse events [18]. Therefore, recent guidelines recommend the use of insulin to maintain the perioperative blood glucose level below 180 mg/dl (10.0 mmol/L) [11] [19]. CPB can profoundly impact the prognosis of diabetic patients undergoing CABG through an obvious disturbance of glucose metabolism [20]. The proposed mechanisms of this disturbance include exacerbated oxidative stress and inflammation, altered hemodynamics, electrolyte disturbances and lowered body temperature [21]. Therefore, personalized glycemic control is needed by diabetic patients undergoing On-Pump CABG.

In our study, strict glucose control, compared with moderate glucose control, was associated with higher rates of the composite endpoint, in-hospital mortality and major vascular complications, and was associated with adverse outcomes after adjusting for confounders. The use of CPB can exacerbate insulin resistance in diabetic patients. Insulin resistance is very common in diabetic patients, resulting in relatively poor auto-regulation of blood glucose levels. On-Pump CABG, and in particular cardiac arrest-resumption, can stimulate release of inflammatory cytokines, exacerbating insulin resistance while affecting glucose metabolism in cardiac tissues [22], ultimately inducing a rapid increase in blood glucose. Consequently, patients undergoing On-Pump CABG may not tolerate strict blood glucose control during the perioperative period. This finding suggests that the target blood glucose level should be adjusted individually for diabetic patients undergoing Om-Pump CABG and a relatively higher blood glucose level may be beneficial for this population.

### Blood glucose fluctuations and in-hospital adverse outcomes

Current studies demonstrate that glycemic variability can be used not only to monitor blood glucose fluctuations, but also to predict the development of cardiovascular complications in diabetic patients [23]. Our study is the first, to our knowledge, to note the impact of blood glucose fluctuations, characterized by LAGE, on in-hospital adverse events in On-pump CABG patients with diabetes. LAGE can be used to effectively characterize glycemic variability, which is associated with beta-cell function in Chinese patients with type 2 diabetes [24]. In our study, we found that patients with relatively greater blood glucose fluctuations (LAGE ≥ 4.4 mol/L) had higher rates of the composite endpoint and major vascular complications in both the moderate- and strict-control groups.

There is a U-shaped correlation between adverse cardiovascular events and blood glucose level in patients with coronary artery diseases [25]. Insulin infusions are widely endorsed to quickly achieve and maintain glucose control, but iatrogenic fluctuations in blood glucose caused by inappropriate insulin therapies in patients with type 2 diabetes may exacerbate oxidative stress and endothelial dysfunction, thereby increasing the risk of macrovascular complications [26]. Notably, a relatively liberal glycemic level is recommended in diabetic patients after On-Pump CABG, especially those transferred to ICU, as these patients tend to have hyperglycemic tolerance [27]. Free fatty acids increase during CPB as a function of heparin-induced lipoprotein lipase. Increased levels of free fatty acids can be toxic to an ischemic heart and result in arrhythmias. Glucose, as the main energy source for the heart during CPB, is known to reduce free fatty acid levels, therefore reducing the cardiotoxic effect [28].

In our study, we found that blood glucose control could impact the in-hospital clinical outcomes by inducing blood glucose fluctuations. These data suggest that continuous blood glucose monitoring is necessary for diabetic patients after On-Pump CABG, especially in patients receiving insulin treatment. Future studies are needed to identify a more precise blood glucose control level to reduce blood glucose fluctuations.

### Strengths and limitations

Our study is the first attempt to explore the association between different blood glucose control levels and in-hospital adverse outcomes. These data should help to improve clinical glycemic management in diabetic patients undergoing CABG. We found that tight glycemic control (< 7.8 mmol/L) increased the risk of adverse outcomes during hospitalization. Also, this is the first study to show that blood glucose fluctuations at different glucose control levels can impact in-hospital clinical outcomes in diabetic patients undergoing On-Pump CABG. Our study provides evidence for appropriate blood glucose levels in this population, which could be helpful for comprehensive post-operative management of diabetic patients. Nevertheless, there are some limitations in this study. First, risk factors related to the CPB procedure, such as the duration of extracorporeal circulation and the depth of anesthesia, which might affect patients’ postoperative blood glucose, were not explored in this study. Second, this study was retrospective and the study population could not be fully matched. A larger and more prospective cohort study is needed to investigate more precise blood glucose control levels for diabetic patients undergoing On-Pump CABG.

## Conclusion

For patients undergoing On-pump CABG, strict glycemic control (BG < 7.8 mmol/L) was associated with an increased risk of both in-hospital mortality and major vascular complications. Meanwhile, LAGE ≥ 4.4 mmol/L was an independent risk factor for the composite endpoint and major vascular complications in patients in both the strict- and moderate-control groups (BG 7.8–9.9 mmol/L).

## Supplementary Information

**Additional file 1: Table S1.** Clinical Outcomes after CPB-CABG by Glycemic Control.

## Data Availability

The data that support the findings of this study are available from the corresponding author upon reasonable request.

## Abbreviations

CABG: Coronary bypass grafting
CPB: Cardiopulmonary bypass
CI: Confidence interval
ICU: Intensive care unit
LAGE: Largest amplitude of glycemic excursion
MBG: Mean blood glucose
OR: Odds ratio
SD: Standard deviation
