# Supplementary material for: Effect of glycemic control and glucose fluctuation on in-hospital adverse outcomes after on-pump coronary artery bypass grafting in patients with diabetes: a retrospective study
Source: Diabetol Metab Syndr. 2023 Feb 14;15:20. doi: 10.1186/s13098-023-00984-4 (PMC9930270; doi:10.1186/s13098-023-00984-4)
Supplement: Supplementary file 1 — Additional file 1: Table S1. Clinical Outcomes after CPB-CABG by Glycemic Control. [file 13098_2023_984_MOESM1_ESM.docx]

**Additional file**

**Table S1 Clinical Outcomes after CPB-CABG by Glycemic Control**

| **Clinical Outcome（%）** | **All Patients**  **(n=1638)** | **Strict Control**  **(n=100)** | **Moderate Control**  **(n=1050)** | **Liberal Control (n=488)** |
| --- | --- | --- | --- | --- |
| **The Composite Endpoint** | 126(7.7) | 14(14.0)* | 73(6.9) | 39(8.0) |
| **MajorVascular Complication** | 122 | 12(12.0)* | 71(6.8) | 39(8.0) |
| **In-hospital Mortality** | 30 (1.8) | 9 (9.0)* | 15 (1.4) | 39 (8.0) |
| **Acute Myocardial Infraction** | 66 (5.4) | 3 (3.0) | 41 (5.3) | 22 (6.0) |
| **Stroke** | 24 (1.5) | 1 (1.0) | 17 (1.6) | 6 (1.2) |
| **Acute Kidney Injury** | 53 (3.2) | 8 (8.0) | 29 (2.8) | 16 (3.3) |
| * P<0.05 is significant, comparing with the moderate control subgroup | | | | |
